# Potential mechanism of tea for treating osteoporosis, osteoarthritis, and rheumatoid arthritis

**DOI:** 10.3389/fmed.2024.1289777

**Published:** 2024-02-14

**Authors:** Xinyu Xie, Jiehui Fu, Weiying Gou, Yifei Qin, Dingzhen Wang, Zuer Huang, Lili Wang, Xihai Li

**Affiliations:** ^1^Academy of Integrative Medicine, Fujian University of Traditional Chinese Medicine, Fuzhou, China; ^2^Fujian Key Laboratory of Integrative Medicine on Geriatrics, Fujian University of Traditional Chinese Medicine, Fuzhou, China; ^3^Department of Sports Medicine (Orthopedics), Fujian University of Traditional Chinese Medicine Subsidiary Rehabilitation Hospital, Fuzhou, China; ^4^College of Integrative Medicine, Fujian University of Traditional Chinese Medicine, Fuzhou, China

**Keywords:** *Camellia sinensis*, osteoporosis, osteoarthritis, rheumatoid arthritis, cyberpharmacology, molecular docking

## Abstract

Osteoporosis (OP), osteoarthritis (OA), and rheumatoid arthritis (RA) are common bone and joint diseases with a high incidence and long duration. Thus, these conditions can affect the lives of middle-aged and elderly people. Tea drinking is a traditional lifestyle in China, and the long-term intake of tea and its active ingredients is beneficial to human health. However, the mechanisms of action of tea and its active ingredients against OP, OA, and RA are not completely elucidated. This study aimed to assess the therapeutic role and related mechanisms of tea and its active ingredients in OP, OA, and RA. Moreover, it expanded the potential mechanisms of tea efficacy based on network pharmacology and molecular docking. Results showed that tea has potential anti-COX properties and hormone-like effects. Compared with a single component, different tea components synergize or antagonize each other, thereby resulting in a more evident dual effect. In conclusion, tea has great potential in the medical and healthcare fields. Nevertheless, further research on the composition, proportion, and synergistic mechanism of several tea components should be performed.

## 1 Introduction

Musculoskeletal disorders, such as osteoporosis (OP), osteoarthritis (OA), and rheumatoid arthritis (RA), are prevalent health issues with substantial global impact. Epidemiological data have revealed the widespread development of these bone-related conditions across various age groups (1–3). OP is defined as bone brittleness, which is associated with fracture susceptibility (4). OA is characterized by cartilage degradation, leading to pain and impaired mobility (5). RA is a condition causing joint deformities and other organ pathologies (6). Current pharmacological therapies (such as bisphosphonates for OP, non-steroidal anti-inflammatory drugs for OA, and disease-modifying antirheumatic drugs for RA) do not provide a fundamental solution to issues with significant safety risks (7–9). Therefore, safer and more promising alternatives should be investigated.

Tea (*Camellia sinensis*), a traditional beverage, has several benefits for the human body (10, 11). In a randomized placebo-controlled trial, postmenopausal women with osteopenia who received green tea polyphenols exhibited better bone health (12). In another randomized controlled trial involving 50 participants, individuals who supplemented their diclofenac tablets with green tea had significantly lower pain scores, as measured using the visual analog scale, and better OA physical function scores compared with controls (13). In addition, a case–control study has revealed that high tea consumption can have a protective effect on smokers and individuals with anti-citrullinated protein autoantibody-positive RA (14). Nonetheless, the comprehensive roles of tea and its extracts in OP, OA, and RA must be systematically elucidated.

This review aimed to examine the current therapeutic mechanisms of tea and its extracts against OP, OA, and RA. In addition, the key active components and target proteins of tea were identified via computer simulations, thereby providing a theoretical foundation for its potential medical and healthcare applications.

## 2 Effects of tea against OP, OA, and RA

Tea has remarkable performance due to its antioxidant and anti-inflammatory properties. Therefore, it can be a promising candidate when used as a novel anti-inflammatory or antioxidant agent (15, 16). Furthermore, contemporary research can provide substantial evidence supporting the role of tea in preventing various diseases, particularly joint diseases, inhibiting disease progression, and promoting pain relief (17, 18). Catechins, which encompass (+)-catechin (C), (−)-epigallocatechin (EC), (−)-gallocatechin (GC), (−)-epigallocatechin gallate (ECG), (−)-epigallocatechin (EGC), and (−)-epigallocatechin-3-gallate (EGCG), are the primary components of tea (19, 20). These compounds, which are naturally consumed via tea consumption, play an essential role in maintaining bodily health. Current research focuses on the anti-inflammatory and antioxidant activities of tea and its components. That is, they promote osteoblast growth and inhibit osteoclast formation, thereby counteracting OP. In addition, these activities inhibit chondrocyte damage and synovial inflammation, which then promote resistance to OA and RA.

In this context, the current study primarily aimed to provide an overview of the potential mechanisms underlying the effects of tea against OP, OA, and RA.

### 2.1 Studies of tea treating osteoporosis

OP, characterized by low bone mass (osteopenia) and deterioration of bone microarchitecture, leads to compromised bone strength and an increased risk of fractures (21). There are several effective strategies against OP. These include the maintenance of bone homeostasis, which enhances bone density, microarchitecture, and strength and reduces the risk of OP and associated fractures (22–25). We gathered clinical studies of tea-treating OP, which are summarized in Table 1. A study showed that in postmenopausal women, additional intake of green tea polyphenol supplementation can improve the serum and urinary levels of oxidative damage biomarkers (31). Furthermore, it can increase the production of bone formation markers and improve bone turnover rates (12). In particular, the intake of active ingredients such as tea water extracts and tea polyphenols leads to significant improvements in bone mineral density, microstructure deterioration, and biological properties in ovariectomized or orchiectomized rats (32–35).

**Table 1:** Clinical trial of tea or components treating osteoporosis.

Tea/Compounds	Subject	Mounts	Age range	Effect	Source
Green tea polyphenols	Women with osteoporosis	171	/	Effective	(12)
Green tea, oolong tea, and black tea	Men and women	25,045	Aged 38–86 years	Effective in women but not in men.	(23)
Tea	Men and women	42,742	Aged 45–74 years	Effective	(25)
Tea	Women	1,377	Aged <80 years	Effective	(26)
Tea	Women with osteoporosis	91,465	Aged 50–79 years	Effective	(27)
Tea	Postmenopausal women	724	Mean age was 57.6 ± 9.6 years	May have a positive effect on BMD but was not found to be a statistically significant factor.	(28)
Oolong tea	Postmenopausal women	476	Aged 40 to 88 years	Effective	(29)
Tea and flavonoid	Women	1,188	Aged >75 years	Effective	(30)

Maintaining bone homeostasis is important for addressing OP by regulating osteoblasts and mesenchymal stem/stromal cells to achieve a balance between bone formation and resorption (36–38). Tea extracts have antioxidant effects that enhance osteoclastogenesis, improve cell survival, and mitigate inflammation (39). Moreover, tea extracts exhibit potent phytoestrogenic effects by upregulating ESR1 expression (40, 41). EGC significantly upregulated the expression of key markers of bone formation, including Runt-related transcription factor 2 (RUNX2), alkaline phosphatase, osteonectin, and osteopontin (42). (−)-Epiafzelechin and (−)-epicatechin promote osteoblast proliferation and differentiation via their antioxidant properties (43). In addition, (−)-epicatechin gallate stimulates osteoblast differentiation by activating the PDZ-binding motif (TAZ) and RUNX2 (44). EGCG has antioxidant effects via the Nrf2 pathway, thereby protecting osteoblasts from apoptosis and attenuating bone microstructure deterioration (45). Theaflavin-3,3′-digallate activates several signaling pathways, including the tumor necrosis factor-α (TNF-α)-inhibited mitogen-activated protein kinase (MAPK), Wnt/β-catenin, and BMP/Smad pathways. This mechanism ultimately promotes the transcription of osteogenesis-associated factors such as RUNX2 and Osterix, leading to osteoblast differentiation and maturation (46). Furthermore, various tea extracts and tea polyphenols inhibit osteoclast formation, with EGCG being the most effective (47–51). Notably, EGCG downregulates the expression of NFATc1, directly binds to RANK, blocks the interaction between RANK and RANKL, and inhibits multiple pathways, including the HO-1-HMGB1-AGE pathway, nuclear factor kappa B (NF-κB) pathway, MAPK signaling pathway, and RANK/RANKL/OPG pathway, ultimately reducing osteoclast formation (52–54). In addition, tea extracts and EGCG enhance the osteogenic differentiation capacity of stem cells (55–57), thereby underscoring the anti-osteoporotic potential of tea and its compounds.

### 2.2 Studies of tea treating osteoarthritis

OA is a prevalent chronic joint disorder primarily characterized by joint cartilage degeneration, synovial inflammation, and pain. Moreover, it is often associated with the aging process (63, 64). We gathered clinical studies of tea treating OA and summarized in Table 2. Several studies have reported that high green tea intake is associated with a low incidence of OA (65, 66). In a short-term randomized, double-blind pilot study, a mixture of baicalin and catechin was as effective as naproxen in controlling the signs and symptoms of knee OA (67). Green tea has anti-inflammatory properties (58). Hence, tea and its extracts can mitigate the pathological progression of OA by decreasing the expression of inflammatory factors, including interleukins and matrix metalloproteinases, in the articular cartilage and synovium, thereby suppressing the inflammatory response.

**Table 2:** Clinical trial of tea or components treating osteoarthritis.

Tea/Compounds	Subject	Mounts	Age range	Effect	Source
Green tea	Men and women	50	Aged 40 to 75	Effective	(13)
Anti-inflammatory diet with green tea	Men and women	18	Between 20 and 80	Effective	(58)

Research on the effects of tea beverages on OA dates has been conducted since 1991 (68). Haqqi et al. have made significant contributions by focusing on the pharmacodynamic mechanisms of tea and its components for treating OA. They discovered that tea polyphenols when added to water, can be effective in preventing the development and progression of arthritis (69). Furthermore, they found that EGCG can reduce the expression and activity of various factors, including cyclooxygenase-2 (COX-2), nitric oxide synthase-2 (NOS-2) (70), matrix metalloproteinase (MMP)-1, MMP-13 (71), and TNF-α (72). In addition, EGCG can globally suppress the inflammatory response in human chondrocytes, possibly via the inhibition of NF-κB and c-Jun N-terminal kinase (JNK)-MAPK activation (73–75). A previous study has consistently revealed the protective effect of EGCG against OA (76). Furthermore, its mechanisms have been found to be involved in various processes such as microRNA regulation (e.g., microRNA-140-3p, microRNA-199a-3p, and microRNA-29b) (77–79) and oxidative stress (77, 78). In addition to its chondroprotective effects, EGCG alleviates synovial inflammation (80). Another study has explored the role of other tea components, such as theaflavin-3,3′-digallate (81) and theaflavin (82), both of which have the ability to inhibit cartilage damage. In recent years, previous studies have focused on enhancing the anti-inflammatory effects of tea and its components. Several studies have improved the efficacy of EGCG in the cartilage and synovium by introducing novel materials or altering the mode of application. These mechanisms involve the modulation of autophagy, the production of reactive oxygen species, mitochondrial repair, and synovial macrophage polarization (83–86).

### 2.3 Studies of tea treating rheumatoid arthritis

In a review published in 2001, the authors proposed that green tea can be a prophylactic agent against chronic inflammatory diseases, including RA (87). We gathered Clinical trial of tea or components treating rheumatoid arthritis in Table 3. A case–control study showed that consuming more than one cup of green tea per month can have a preventive effect against RA (88). Maintaining a daily intake of 4–6 cups of green tea over a period of up to 6 months has a positive effect on RA disease activity in patients with RA (59). In addition, a Swedish case–control study showed that heavy tea consumption has a protective effect against RA in smokers and anti-citrullinated protein autoantibody-positive individuals (14). Numerous experiments have revealed that tea water extracts or the polyphenolic components of tea can reduce RA in experimental animals. The investigation of its mechanism has predominantly revolved around its antioxidant and anti-inflammatory properties.

**Table 3:** Clinical trial of tea or components treating rheumatoid arthritis.

Tea/Compounds	Subject	Mounts	Age range	Effect	Source
Green tea	Subjects with rheumatoid arthritis	120	Mean age of (60.7 ± 2.53 years)	Effective	(59)
Aqueous green tea extract	Subjects with/without rheumatoid arthritis	130	Aged >40 years	Effective	(60)
Epigallocatechin gallate	Subjects with/without rheumatoid arthritis	50	Aged between 25 and 60 years	Effective	(61)
Tea	Subjects with rheumatoid arthritis	662	/	Effective	(62)

Various tea-related ingredients, including tea aqueous extract (89), catechin (90), EGCG, and gallic acid (91), are significantly effective in alleviating RA symptoms. The imbalance between oxidation and reduction is an important mechanism in the development of RA (92). Reactive species oxidize cellular biomolecules, leading to DNA damage (93). Therefore, reducing oxidative stress in RA is an effective therapeutic strategy (94). Physiological antioxidant enzymes such as superoxide dismutase (SOD), glutathione (GSH), and peroxiredoxins counteract the possible damaging effects of these reactive species by scavenging or neutralizing free radicals and oxidizing substances. Research has shown that green tea extract can increase the SOD and GSH levels while decreasing the levels of lipid peroxides (LPO), nitric oxide (NO), and PGE2 in a rat RA model. Hence, it can be beneficial in both the liver and brain (95, 96). EGCG-fed mice exhibited higher levels of heme oxygenase-1 (HO-1) and nuclear factor erythroid2-related factor 2 (Nrf2) (97, 98), and the significant activation of HO-1 and Nrf2 has anti-arthritic effects (99). In addition to their antioxidant effects, tea and its compounds have significant anti-inflammatory effects. Sabrina Fechtner has revealed that EGCG, epigallocatechin (EGC), and EC occupy the active site of the TAK1 kinase domain, with EGCG being the most dominant, interfering with the IL-1β signaling pathway that regulates the expression of IL-6, IL-8, and Cox-2 in primary human RA synovial fibroblasts (100). Another study revealed that EGCG targets TAK1 for treating RA by inhibiting TAK1 phosphorylation at Thr (184/187), suppressing K(63)-linked autoubiquitination of TRAF6, and enhancing proteasome-associated deubiquitinase expression to rescue proteins from proteasomal degradation (101). In addition, green tea extract and EGCG modulate the production of chemokine (102) and immune cells (97), leading to RA improvements.

## 3 Assessment of the potential active components of tea in OP, OA, and RA

Since tea performed well not only in OP but also in OA and RA, searching for targets in OP, OA, and RA may have a practical meaning in providing guidance for the prevention and control of OP, OA, and RA. With the help of bioinformatics analysis methods, we summarized the ingredients of tea (Table 4) and discovered the relationship between tea ingredients and diseases. We screened the genes of OP, OA, and RA related to tea ingredients and then performed Gene Ontology (GO) analysis and enrichment analysis of the Kyoto Encyclopedia of Genes and Genomes (KEGG) pathway.

**Table 4:** Summary of potential active components from tea.

Name	InChIKey	Source
(−)-Catechin	PFTAWBLQPZVEMU-HIFRSBDPSA-N	(90–93, 95)
(−)-Catechin gallate	LSHVYAFMTMFKBA-PZJWPPBQSA-N	(91, 92, 94, 96)
(−)-Epicatechin	PFTAWBLQPZVEMU-UKRRQHHQSA-N	(14, 90–97)
(−)-Epicatechin-pentaacetate	BKYWAYNSDFXIPL-JWQCQUIFSA-N	(14, 91, 92)
(−)-Epigallocatechin-3-gallate	WMBWREPUVVBILR-WIYYLYMNSA-N	(14, 90–95, 97)
(−)-Gallocatechin gallate	WMBWREPUVVBILR-GHTZIAJQSA-N	(14, 91, 92, 96, 97)
(+)-Catechin	PFTAWBLQPZVEMU-DZGCQCFKSA-N	(14, 90–96)
(+)-Cycloolivil	KCIQZCNOUZCRGH-VOBQZIQPSA-N	(90, 91)
(+)-Epicatechin	PFTAWBLQPZVEMU-ZFWWWQNUSA-N	(90–93, 95, 97)
2-Formylpyrrole	ZSKGQVFRTSEPJT-UHFFFAOYSA-N	(14, 91, 92)
2-Phenylbutenal	DYAOGZLLMZQVHY-MBXJOHMKSA-N	(14, 92)
Aids214634	CICMVLOHBZPXIT-WNISUXOKSA-N	(14)
alpha-Cadinol	LHYHMMRYTDARSZ-BYNSBNAKSA-N	(14)
Astragalin	JPUKWEQWGBDDQB-QSOFNFLRSA-N	(92)
beta-Carotene	OENHQHLEOONYIE-JLTXGRSLSA-N	(14, 97)
Betulinic acid	QGJZLNKBHJESQX-FZFNOLFKSA-N	(14)
Caffeine	RYYVLZVUVIJVGH-UHFFFAOYSA-N	(14, 90–92, 96)
cis-Jasmone	XMLSXPIVAXONDL-PLNGDYQASA-N	(94)
Citral	WTEVQBCEXWBHNA-JXMROGBWSA-N	(14, 95)
Citric acid	KRKNYBCHXYNGOX-UHFFFAOYSA-N	(89, 94, 95)
delta-Terpineol	SQIFACVGCPWBQZ-UHFFFAOYSA-N	(14)
Diosmetin	MBNGWHIJMBWFHU-UHFFFAOYSA-N	(14)
Ellagic acid	AFSDNFLWKVMVRB-UHFFFAOYSA-N	(90, 91, 95, 97)
Epiafzelechin	RSYUFYQTACJFML-UKRRQHHQSA-N	(94, 95)
Epicatechin gallate	LSHVYAFMTMFKBA-FPOVZHCZSA-N	(89, 92, 95–97)
Epigallocatechin	XMOCLSLCDHWDHP-IUODEOHRSA-N	(14, 90, 92, 95–97)
Folic acid	OVBPIULPVIDEAO-LBPRGKRZSA-N	(14, 90–94)
Gallic acid	LNTHITQWFMADLM-UHFFFAOYSA-N	(90, 94–96)
Gallocatechin	XMOCLSLCDHWDHP-SWLSCSKDSA-N	(14, 92, 95–97)
Geraniin	JQQBXPCJFAKSPG-SVYIMCMUSA-N	(14, 92)
Hirsutrin	OVSQVDMCBVZWGM-QSOFNFLRSA-N	(92, 94, 95)
Indole	SIKJAQJRHWYJAI-UHFFFAOYSA-N	(87, 94)
Isomyricitrin	FOHXFLPXBUAOJM-LIBJPBHASA-N	(14)
Isovitexin	MYXNWGACZJSMBT-VJXVFPJBSA-N	(94, 95)
Kaempferitrin	PUPKKEQDLNREIM-SLVXTXDOSA-N	(90, 94–96)
Kaempferol 3-O-glucorhamnoside	SOSLMHZOJATCCP-FPRKOELSSA-N	(90)
Kaempferol 3-O-rhamnoside	SOSLMHZOJATCCP-LYHQQHOMSA-N	(90, 93)
Kaempferol-3-galactoside	JPUKWEQWGBDDQB-DTGCRPNFSA-O	(90)
Kaempferol-3-O-glucuronide	FNTJVYCFNVUBOL-VFKUPZNOSA-N	(90)
L-Epicatechin gallate	LSHVYAFMTMFKBA-TZIWHRDSSA-N	(14, 90, 95)
L-Phenylalanine	COLNVLDHVKWLRT-QMMMGPOBSA-N	(9, 39, 96)
Myricetin-3-O-beta-D-galactopyranoside	FOHXFLPXBUAOJM-MRBQYWCKSA-N	(90, 93, 95)
Myricetin-3-O-beta-D-glucopyranoside	FOHXFLPXBUAOJM-FVNGHLGHSA-O	(90, 93)
Naringin	DFPMSGMNTNDNHN-ZPHOTFPESA-N	(93)
Nicotiflorin	RTATXGUCZHCSNG-QHWHWDPRSA-N	(14)
Nicotinic acid	PVNIIMVLHYAWGP-UHFFFAOYSA-N	(14)
Oleanolic acid	MIJYXULNPSFWEK-GTOFXWBISA-N	(14)
Petunidin	BLBZAMLPGFAHFX-UHFFFAOYSA-N	(90, 91, 93)
Phenethyl isothiocyanate	IZJDOKYDEWTZSO-UHFFFAOYSA-N	(14, 92)
Procyanidin B1	XFZJEEAOWLFHDH-UKWJTHFESA-N	(92–95)
Procyanidin B2	XFZJEEAOWLFHDH-NFJBMHMQSA-N	(92–95)
Quercetin,3-O-rutinoside	IKGXIBQEEMLURG-BKUODXTLSA-O	(90, 95)
Quercitrin	OXGUCUVFOIWWQJ-HQBVPOQASA-N	(90, 93, 95, 96)
Quinic acid	AAWZDTNXLSGCEK-WYWMIBKRSA-N	(90–93)
Rutin	IKGXIBQEEMLURG-NVPNHPEKSA-N	(93–96)
Theobromine	YAPQBXQYLJRXSA-UHFFFAOYSA-N	(14, 90, 92, 94–96)
Tricin	HRGUSFBJBOKSML-UHFFFAOYSA-N	(14, 92)
Trifolin	JPUKWEQWGBDDQB-DTGCRPNFSA-N	(14, 92)
Tryptophan	QIVBCDIJIAJPQS-VIFPVBQESA-N	(92, 96)
Ursolic acid	WCGUUGGRBIKTOS-GPOJBZKASA-N	(14, 92)
Vicenin-2	FIAAVMJLAGNUKW-VQVVXJKKSA-N	(92, 95, 97)
Xanthine	LRFVTYWOQMYALW-UHFFFAOYSA-N	(14, 92, 97)

### 3.1 Oxidative stress mitigation: unraveling pathways

Due to aging, traumatic injury, or immune dysfunction, various tissue cells are exposed to a range of pathophysiological mediators, including reactive oxygen species (ROS) and reactive nitrogen species (NOS). ROS-mediated stress, by inducing functional impairments in osteoblasts, osteoclasts, chondrocytes, and synovial cells, contributes to the pathological progression of OP, OA, and RA.

**Figure 1:**
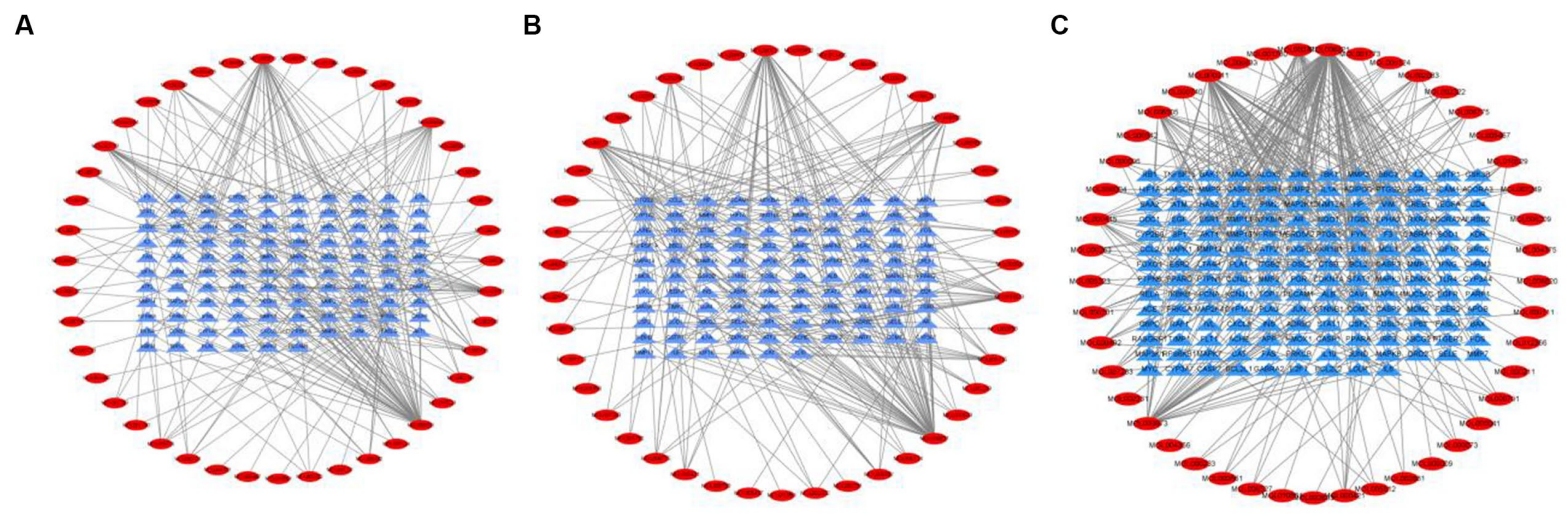
Networks of tea components targeting OP, OA and RA. **(A)** Network of tea treating OP; **(B)** Network of tea treating OA; **(C)** Network of tea treating RA.

**Figure 2:**
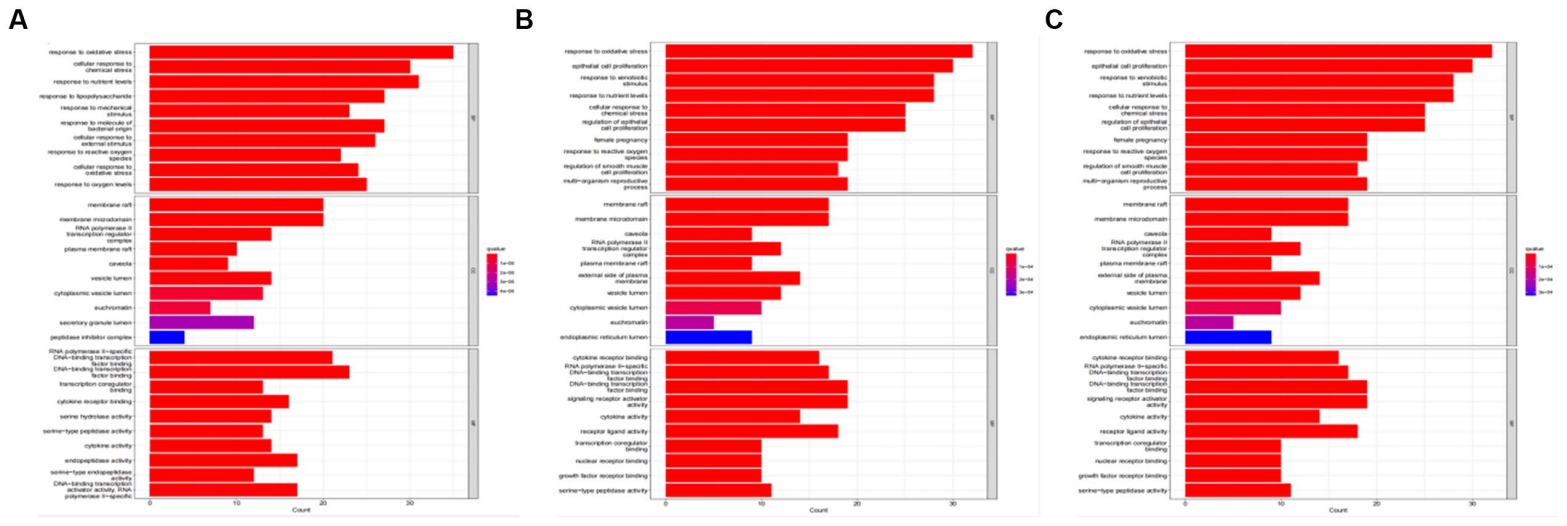
GO analysis of tea targeting OP, OA, and RA. **(A)** GO analysis of tea targeting OP; **(B)** GO analysis of tea targeting OA; **(C)** GO analysis of tea targeting RA.

**Figure 3:**
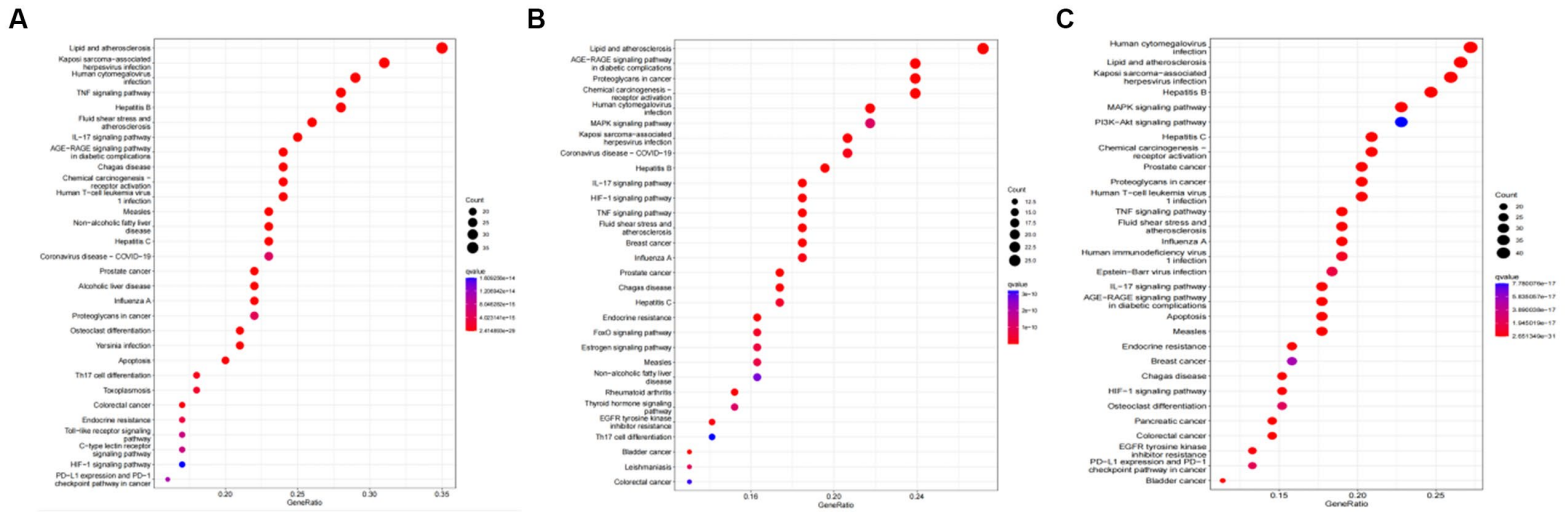
KEGG analysis of tea targeting OP, OA, and RA. **(A)** KEGG analysis of tea targeting OP; **(B)** KEGG analysis of tea targeting OA; **(C)** KEGG analysis of tea targeting RA.

Tea exhibits robust antioxidant effects, a perspective supported by network pharmacology results. The active ingredient–gene target networks of the active ingredients for each disease were mapped (Figure 1). Gene Ontology (GO) enrichment analysis reveals that genes associated with OP, targeted by active components of tea, are primarily enriched in response to oxidative stress (Figure 2A). In the GO analysis results of networks between tea and OA and between tea and RA, genes linked to response to oxidative stress occupy the top positions (Figures 2B,C). The frequency ranking of responses to oxidative stress across the three diseases is detailed in Table 5. Among the 37 tea components targeting genes related to this biological process, EGCG, caffeine, ursolic acid, beta-carotene, and (−)-epicatechin emerge as the top five components. KEGG pathway analysis indicates enrichment in pathways such as lipid and atherosclerosis and the AGE-RAGE signaling pathway in diabetic complications (Figure 3 and Table 6). Although direct evidence of oxidative stress is not explicitly shown in the KEGG results, the enrichment of pathways closely related to oxidative stress responses underscores the pivotal role of tea’s antioxidant action in the treatment of OP, OA, and RA.

**Table 5:** Gene Ontology (GO) enrichment analysis.

Disease	ID	Description	GeneRatio	BgRatio	*p*-value	p.adjust	*q*-value
Osteoporosis	GO:0006979	Response to oxidative stress	32/95	434/18903	6.00E-29	2.21E-25	9.89E-26
Osteoarthritis	GO:0006979	Response to oxidative stress	35/106	434/18903	3.23E-31	1.22E-27	5.36E-28
Rheumatoid arthritis	GO:0006979	Response to oxidative stress	45/165	434/18903	1.01E-35	4.33E-32	1.80E-32

**Table 6:** KEGG pathway analysis of tea treating OP, OA, and RA.

Disease	ID	Description	GeneRatio	*p*-value	p.adjust	*q*-value
Osteoporosis	hsa05417	Lipid and atherosclerosis	25/92	2.06E-19	2.45E-17	7.91E-18
	hsa04933	AGE-RAGE signaling pathway in diabetic complications	22/92	2.04E-23	4.86E-21	1.57E-21
	hsa05205	Proteoglycans in cancer	22/92	2.36E-16	1.41E-14	4.54E-15
	hsa05207	Chemical carcinogenesis - receptor activation	22/92	4.86E-16	2.31E-14	7.47E-15
	hsa05163	Human cytomegalovirus infection	20/92	2.47E-13	3.78E-12	1.22E-12
Osteoarthritis	hsa05417	Lipid and atherosclerosis	35/100	3.32E-31	7.98E-29	2.41E-29
	hsa05167	Kaposi sarcoma-associated herpesvirus infection	31/100	2.98E-27	1.79E-25	5.41E-26
	hsa05163	Human cytomegalovirus infection	29/100	1.06E-22	2.55E-21	7.72E-22
	hsa04668	TNF signaling pathway	28/100	3.77E-30	4.53E-28	1.37E-28
	hsa05161	Hepatitis B	28/100	1.48E-25	5.91E-24	1.79E-24
Rheumatoid arthritis	hsa05163	Human cytomegalovirus infection	43/158	3.68E-32	2.59E-30	8.49E-31
	hsa05417	Lipid and atherosclerosis	42/158	8.35E-32	4.29E-30	1.41E-30
	hsa05167	Kaposi sarcoma-associated herpesvirus infection	41/158	1.71E-32	2.20E-30	7.21E-31
	hsa05161	Hepatitis B	39/158	3.15E-33	8.09E-31	2.65E-31
	hsa04010	MAPK signaling pathway	36/158	5.79E-20	7.09E-19	2.32E-19

### 3.2 Inflammatory modulation: an alternate pathway for tea impact

**Figure 4:**
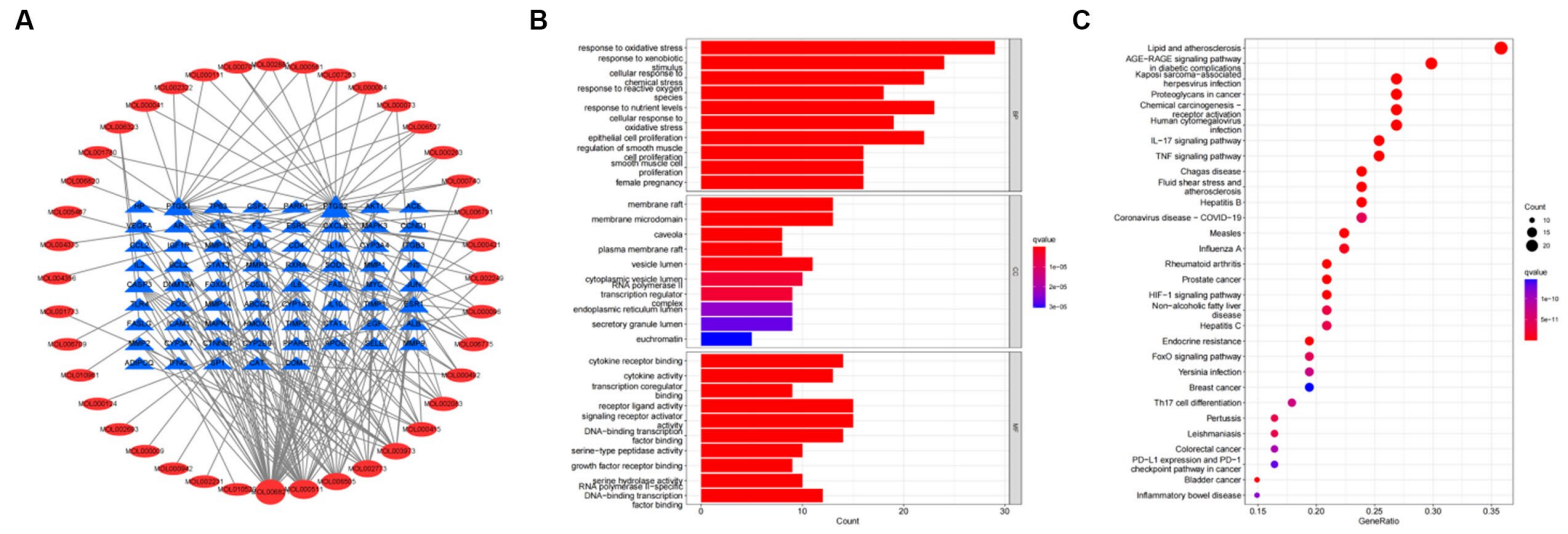
Bioinformatics analysis for tea targeting common genes of OP, OA, and RA. **(A)** Network of tea treating common genes, **(B)** Gene ontology (GO) enrichment analysis; **(C)** Kyoto encyclopedia of genes and genomes (KEGG) pathway enrichment analysis.

To further investigate the therapeutic effects of tea in treating OP, OA, and RA, network pharmacology was applied to analyze the targeted relationship between tea and commonly associated genes. The analysis results not only confirm the involvement of oxidative stress in line with previous findings but also reveal enrichment in pathways such as lipid and atherosclerosis, the AGE-RAGE signaling pathway in diabetic complications, the IL-17 signaling pathway, and the TNF signaling pathway through KEGG analysis (Figure 4 and Table 7). This demonstrates the anti-inflammatory effects of tea, with core genes such as PTGS2, PTGS1, CASP3, JUN, and IL-6 remaining central in these pathways. A total of 36 tea components target genes related to these pathways, with (−)-epigallocatechin-3-gallate, caffeine, ursolic acid, beta-carotene, and (−)-epicatechin ranking as the top five components targeting the highest number of genes. This suggests that these components play a core role in anti-inflammatory action. Importantly, these components also play a significant role in the previously mentioned antioxidative effects. Therefore, we utilized computer-simulated molecular docking to further validate the relationships between these components and core proteins.

**Table 7:** GO and KEGG analyses for tea targeting common genes of OP, OA, and RA.

Analysis type	ID	Description	GeneRatio	BgRatio	*p*-value	p.adjust	*q*-value
GO	GO:0006979	Response to oxidative stress	29/69	434/18903	1.18E-29	3.83E-26	1.50E-26
KEGG	hsa04933	AGE-RAGE signaling pathway in diabetic complications	20/67	100/8644	9.38E-24	2.10E-21	6.12E-22
	hsa05417	Lipid and atherosclerosis	24/67	215/8644	3.30E-22	3.69E-20	1.08E-20
	hsa04657	IL-17 signaling pathway	17/67	94/8644	2.00E-19	1.49E-17	4.35E-18
	hsa04668	TNF signaling pathway	17/67	114/8644	6.32E-18	3.54E-16	1.03E-16
	hsa05142	Chagas disease	16/67	102/8644	2.88E-17	1.29E-15	3.75E-16

**Figure 5:**
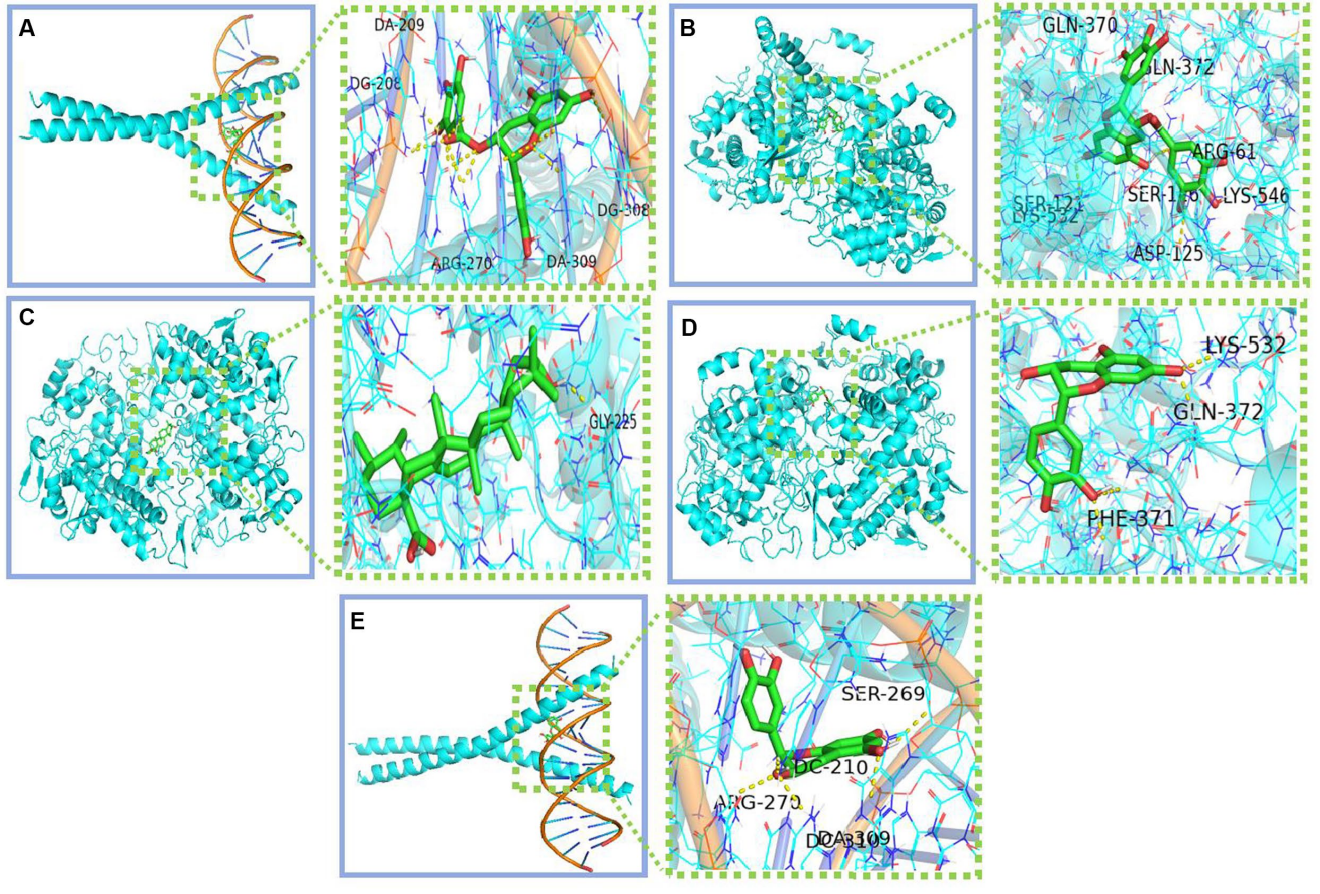
Combinations with affinity below −9 kCal∙mol-1 **(A)** Jun-EGCG; **(B)** PTGS2-EGCG; **(C)** PTGS2-Ursolic acid; **(D)** PTGS2-Epicatechin; **(E)** Jun-Epicatechin.

We conducted molecular docking for the aforementioned components (EGCG, ursolic acid, beta-carotene, (−)-epicatechin, and caffeine) and proteins (PTGS2, PTGS1, CASP3, IL-6, and JUN) with established targeting relationships. The affinity for each combination was below −5 kCal ∙ mol-1. Thus, it has favorable binding activity. Furthermore, most combinations exhibited an affinity below −7 kCal ∙ mol-1, which indicated a robust binding activity (Table 8). Figure 5 shows the combinations featuring hydrogen bonds whose affinity is below −9 kCal∙mol-1.

**Table 8:** Summary of affinity of each combination.

Affinity (kcal/mol)	EGCG	Ursolic acid	Beta-Carotene	(−)-Epicatechin	Caffeine
PTGS2	−10.4	−9.8	−7.9	−9.1	−6.7
PTGS1	/	−7.6	/	−8.0	−6.9
CASP3	−8.7	−8.4	−8.7	−7.7	−5.3
IL-6	−7.2	−8.7	/	−7.3	−5.2
JUN	−11.0	−7.5	−7.7	−9.1	−6.8

## 4 Discussion

The Chinese have been drinking tea for hundreds of years. Therefore, most people believe that tea can reduce the risk of various diseases. With the development of modern medicine, the efficacy and mechanism of action of tea have been comprehensively explored. However, the results of clinical and animal studies are still inconclusive. This review aimed to explore the possibility and mechanism of action of tea for treating OP, OA, and RA by evaluating previous studies and constructing a network for the association between tea and different diseases. Current studies have focused on the anti-inflammatory and antioxidant effects of tea. Tea and its components affect the activation of various enzymes, transcription of inflammation-related genes, and release of inflammatory factors in bone and joint tissues via the Nrf2-related pathway, MAPK pathway, and NF-κB pathway. Moreover, they regulate oxidative stress and inflammation in tissues and cells in OP, OA, and RA.

Network pharmacology results show that PTGS2, PTGS1, CASP3, IL-6, and JUN are the potential targets of tea when regulating OP, OA, and RA. PTGS2 (also referred to as COX-2) and PTGS1 (also known as COX-1) have been extensively and intensively evaluated. COX inhibitors, or non-steroidal anti-inflammatory drugs, inhibit the production of COX-2 and COX-1 to achieve anti-inflammatory, analgesic, and antipyretic effects. In addition, they are commonly used in the treatment of OA and RA (103). The anti-inflammatory and analgesic efficacies of COX inhibitors are significant. However, they also increase the risk of gastrointestinal ulcers, hemorrhage, and renal and cardiovascular adverse events (104). In the molecular target regulatory network of tea and disease (OP, OA, and RA), 31 molecules can bind to PTGS2 targets, and 18 molecules can bind to PTGS1 targets. Therefore, tea can possibly play a role in COX inhibition. However, epidemiological studies have shown that tea consumption reduces the risk of cardiovascular mortality through mechanisms associated with the lowering of lipid levels, mitigation of ischemia/reperfusion injury (105, 106), inhibition of oxidative stress, enhancement of endothelial function, attenuation of inflammation, and protection of cardiomyocyte function (107). The tea polyphenol EGCG exerted a protective effect on patients with 5-aminosalicylic acid and/or azathioprine-refractory ulcerative colitis (108). According to the report, TIMP1, PTGS2, ICAM1, MMP9, IL1B, CXCL8, IL-6, and RELA were identified as hub genes in ulcerative colitis (109), which had been found in the target collection of tea components. A new study, processed by integrating network pharmacology and metabolomics, demonstrated that *Jasminum elongatum* reverses ulcerative colitis in mice via the IκB/p65/COX-2/arachidonic acid pathway (110). The tea aqueous extract inhibited experimentally induced colitis and liver injury in mice (111). Tea and its extracts confer protective effects against alcoholic liver disease, non-alcoholic fatty liver disease, CCL4-induced liver injury, and inflammatory liver damage. The mechanisms underlying these protective effects involve modulation of signaling pathways such as the NF-κB signal pathway, TGFβ/p-ERK/p-Smad1/2 signal pathway, Nrf2 signaling activation, and autophagy restoration (112–117). Tea consumption has been associated with a reduced risk of renal cell carcinoma (118) and improved kidney function in diabetic patients (119). Studies indicate that effective components such as L-theanine, tea polyphenols, and EGCG can ameliorate renal cell damage through the modulation of related pathways, including the AGEs/RAGE signaling pathway (120), CYP450s/ROS/MAPK/NF-κB pathway (121), TGFβ/Smad3 signaling pathway (122), and ferroptosis (123). The abovementioned studies have revealed the potential COX inhibitory effects of tea and its ability to fight diseases such as OP, OA, and RA with minimal cardiovascular, gastrointestinal, hepatic, and renal damage.

Via a network pharmacological analysis, a number of tea ingredients target disease genes in OP, OA, and RA, proving that tea has therapeutic or adjunctive therapeutic effects against OP, OA, and RA. Current clinical studies do not provide clear conclusions. Some reports have shown that tea can be effective in treating OP, OA, or RA. However, there are limitations in terms of the study population, the size of the population, or the quality of the data. Meanwhile, some clinical studies or meta-analyses have revealed that tea consumption does not improve the clinical performance of patients or reduce the risk of OP, OA, or RA (124). Considering the diversity of active ingredients in tea, in addition to ingredients such as EGCG and EC, which play a positive role, other ingredients, such as caffeine, increase the risk of fracture, OP, or OA and can be an influencing factor in the therapeutic effects of tea. Therefore, further studies on the role of tea must be performed.

Studies on effective treatment strategies against OP, OA, and RA are still conducted by the medical community. Tea is rich in various natural compounds that can be used for disease treatment. This study aimed to evaluate the potential mechanisms of action of tea and its related components for treating OP, OA, and RA. These mechanisms of action mainly focus on the antioxidant and anti-inflammatory responses of tea components. In previous experimental studies, tea and tea extracts and their active ingredients mainly acted on OA inflammatory factors to alleviate OA cartilage degeneration. Furthermore, they are mainly used to promote osteoblast growth, inhibit osteoclast formation in OP, and inhibit inflammation mainly via their antioxidant effects in RA. The network pharmacological results revealed targets and pathways not covered by existing experimental studies. Moreover, they were validated by molecular docking. The network pharmacology results showed that tea has an anti-COX capacity, hormone-like properties, and cardiovascular, gastrointestinal, hepatic, and renal protective effects. This is because tea has various components that synergize or antagonize each other, which has a more pronounced dual effect than a single component. In the network pharmacology analysis, we comprehensively collected data on the compounds of tea and did not screen the compounds for bioavailability and drug-like properties so that we could analyze the mechanism of action of tea against OP, OA, and RA analyzed without omission. However, different kinds of tea have different compound compositions; for example, black tea contains theaflavins, thearubigins, and other components, and lower levels of polyphenols compared with green tea, which cannot be represented in network pharmacological analysis results. The network pharmacological analysis results may conceal the specific effects of tea on certain disease genes.

Nevertheless, there is still a need for future research on the composition of tea and the development of standardized tea beverages, which will lead to efficacy studies. Research on the relationship between the use of standardized tea beverages and disease would be helpful to clarify the efficacy of tea. In conclusion, the use of tea has great potential in the medical and healthcare fields.

## Author contributions

XX: Data curation, Formal analysis, Writing – original draft. JF: Investigation, Methodology, Writing – original draft. WG: Conceptualization, Investigation, Writing – original draft. YQ: Investigation, Writing – original draft. DW: Methodology, Writing – original draft. ZH: Formal analysis, Validation, Writing – original draft. LW: Supervision, Writing – review & editing. XL: Funding acquisition, Project administration, Writing – review & editing.
